# A scoping review of registry captured indicators for evaluating quality of critical care in ICU

**DOI:** 10.1186/s40560-021-00556-6

**Published:** 2021-08-05

**Authors:** Issrah Jawad, Sumayyah Rashan, Chathurani Sigera, Jorge Salluh, Arjen M. Dondorp, Rashan Haniffa, Abi Beane

**Affiliations:** 1 National Intensive Care Surveillance-MORU, Borella, Colombo, Western Province 08 Sri Lanka; 2 Department of Critical Care and Graduate Program in Translational Medicine, D’Or Institute for Research and Education, Rio de Janeiro, Brazil; 3 Critical Care, Mahidol Oxford Tropical Medicine Research Unit, Bangkok, Central Thailand 10400 Thailand; 4 Nuffield Department of Clinical Medicine, University of Oxford, Oxford, UK

**Keywords:** Quality indicators, Critical illness, Health system improvement, ICU, Benchmarking, Patient safety

## Abstract

**Background:**

Excess morbidity and mortality following critical illness is increasingly attributed to potentially avoidable complications occurring as a result of complex ICU management (Berenholtz et al., J Crit Care 17:1-2, 2002; De Vos et al., J Crit Care 22:267-74, 2007; Zimmerman J Crit Care 1:12-5, 2002). Routine measurement of quality indicators (QIs) through an Electronic Health Record (EHR) or registries are increasingly used to benchmark care and evaluate improvement interventions. However, existing indicators of quality for intensive care are derived almost exclusively from relatively narrow subsets of ICU patients from high-income healthcare systems. The aim of this scoping review is to systematically review the literature on QIs for evaluating critical care, identify QIs, map their definitions, evidence base, and describe the variances in measurement, and both the reported advantages and challenges of implementation.

**Method:**

We searched MEDLINE, EMBASE, CINAHL, and the Cochrane libraries from the earliest available date through to January 2019. To increase the sensitivity of the search, grey literature and reference lists were reviewed. Minimum inclusion criteria were a description of one or more QIs designed to evaluate care for patients in ICU captured through a registry platform or EHR adapted for quality of care surveillance.

**Results:**

The search identified 4780 citations. Review of abstracts led to retrieval of 276 full-text articles, of which 123 articles were accepted. Fifty-one unique QIs in ICU were classified using the three components of health care quality proposed by the High Quality Health Systems (HQSS) framework. Adverse events including hospital acquired infections (13.7%), hospital processes (54.9%), and outcomes (31.4%) were the most common QIs identified. Patient reported outcome QIs accounted for less than 6%. Barriers to the implementation of QIs were described in 35.7% of articles and divided into operational barriers (51%) and acceptability barriers (49%).

**Conclusions:**

Despite the complexity and risk associated with ICU care, there are only a small number of operational indicators used. Future selection of QIs would benefit from a stakeholder-driven approach, whereby the values of patients and communities and the priorities for actionable improvement as perceived by healthcare providers are prioritized and include greater focus on measuring discriminable processes of care.

**Supplementary Information:**

The online version contains supplementary material available at 10.1186/s40560-021-00556-6.

## Background

Critical illness, including (but not limited to) care for the sickest surgical, trauma, and communicable diseases patients, causes an enormous health and economic burden globally. Patients with critical illness are at high risk for poor outcomes and often require intensive care unit (ICU) admission [1]. Specialties synonymous with critical care, traumatic brain injury, infectious diseases, and perioperative care have all benefited from high-quality clinical trials informing treatment and outcomes, and have all included critically ill populations [2–7]. However, large differences in daily ICU practice and patient outcomes remain, with excess morbidity and mortality following critical illness increasingly attributed to potentially avoidable complications occurring as a result of complex ICU management [8, 9] As a consequence, research has increasingly focused on strategies to improve the effectiveness of common interventions synonymous with ICU management, in an effort to reduce avoidable harm, reduce mortality, and promote quality of life following recovery [1, 10–12].

Routine quality measurement using appropriate indicators can guide care improvement, for example, through identifying existing good practice, and evaluating strategies aimed at targeting sub optimal care. The potential of quality indicators to improve care has already been demonstrated in other clinical areas including maternal and child health, and in the management of sepsis and stroke patients [10, 13–15]. In parallel, clinically facing registries for critical care are expanding internationally. Registries are increasingly seen as a tool to enable the evaluation of existing care by systematically capturing quality of care indicators used to benchmark and compare performance [1, 8, 9]. However, existing indicators of quality for intensive care are derived almost exclusively from relatively narrow subsets of the ICU patient population selected by experts working in high-income healthcare systems. Despite many indicators of quality being developed and advocated for as a measure of performance, the exact number and level of scientific evidence of these indicators remains unclear. Furthermore, there is potential for wide variation in the measurement and reporting of indicators of quality internationally. Such heterogeneity of definition and of measurement impedes utility of QIs for both replicable evaluation of performance over time within an institution, and benchmarking of performance between units. Similarly, an absence of literature on the challenges of “real-world” implementation of indicators, perhaps suggestive  of the variation of definitions of QIs in use, further hinders those seeking to evaluate and improve care from identifying meaningful measures of quality and using them to drive practice and policy change [8, 12, 15, 16]. To enable global registry networks, such as CRIT Care Asia [17], which aims to support communities of practice to measure existing critical care performance and achieve actionable improvement, greater understanding is needed of the indicators of quality currently being used internationally, their evidence base, and the barriers to measurement and reporting. This review aimed to scope the literature on indicators currently being used to evaluate quality of care in intensive care units, map the indicators' evidence base, and describe the variances in both their definition and measurement. In addition, the reviewers summarized challenges of implementation of the indicators and relevant advantages of measurement of the indicators on ICU practice as described in the literature.

## Methods

### Search strategy

Relevant articles were identified by searching the following databases: MEDLINE, EMBASE, CINAHL, Cochrane Database of Systematic Reviews, Cochrane Database of Abstracts of Reviews of Effects, and Cochrane Central Register of Controlled Trials from the earliest available date through to January 31st, 2019. Searches were performed with no language of publication restrictions. Combinations of the following search terms were used**:** *critical care, ICU, intensive care, quality indicator, quality assurance, quality control, benchmarking, performance improvement, quality measure, best practice, and audit, registry, electronic database, surveillance system.* The Cochrane Library was searched using the search term *critical care*. To increase the sensitivity of the search strategy, we also searched the grey literature. This search included identifying and searching websites of relevant critical care societies which have associated registry networks (ICNARC [18], SICSAG [19], ANZICS [20], EpiMed [21], ESICM [22], ICS [23], JSICM [24]). Appropriate wildcards were used in all searches to account for plural words and variations in spelling. Additional articles were identified by searching the bibliographies of those articles identified in the searches and contacting experts in the field of ICU registries.

### Article selection

We selected all articles that identified or proposed one or more QIs to evaluate the quality of care used to evaluate ICU care through registries. For this study, a QI was defined as “a performance measure that compares actual care against an ideal criteria,” in order to help assess quality of care [15]; minimum inclusion criteria was a description of one or more QIs designed to evaluate clinical performance in intensive care. This included measures associated with admission to the ICU, and outcomes following discharge from the ICU within the same hospital admission. Covidence (an online review tool) was used to collate and curate the stages of the literature review [25].

### Article review

Eligible articles were identified using a two phase process: a published method of the Joanna Briggs Institute scoping review framework [26]. In the first phase, two reviewers (SR and CS) independently reviewed the titles and abstracts of retrieved publications and selected relevant articles for possible inclusion in the review. Disagreements between the two reviewers were discussed, and, if agreement could not be reached, the article was retained for further review by AB [11, 26].

In the second phase, the full texts of the remaining articles were independently reviewed by the same two reviewers using a checklist to determine eligibility criteria. Disagreements between the two assessors were discussed, and a third author was consulted if agreement could not be reached [26]. Reviewers were not masked to author or journal name [11]. Two reviewers independently reviewed all full-text articles that satisfied the minimum inclusion criteria and extracted data using a standardized format. Extracted information included (i) QI definition, (ii) variables and guidance for measuring the QI, (iii) modality and frequency of measurement, and (iv) level of evidence [10]. The level of evidence underpinning the definition and application of the indicator was graded using a published classification of evidence for practice guidelines [27]. Quality indicators were classified using the three components of health care quality proposed by the High Quality Health Systems (HQSS) framework [13]: foundation (which includes human resources and governance structures), processes (encompassing measures of safety and timeliness inalongside patient and user experience), and quality impacts (which extends beyond mortality to quality of recovery and life and social economic welfare). The indicators were also categorized as pre, in, or post ICU. Reviewers further judged whether QIs were operational (yes vs. no) and appraised the literature for barriers and enablers to operationalizing or actioning the QIs which were described. Disagreements in assessment and data extraction were resolved by reviewer consensus, and if agreement could not be reached, the article was independently reviewed by a third reviewer (AB). QIs were summarized as counts and proportions using the packages “highcharter” and “epiDisplay” in R statistical software, version 4.0.2 [28]. The protocol for this study follows the guidelines for Preferred Reporting Items for Systematic Reviews and Meta-Analyses extension for Scoping Reviews (PRISMA-ScR) included as an additional file [see Additional File 1].

## Results

**Fig. 1:**
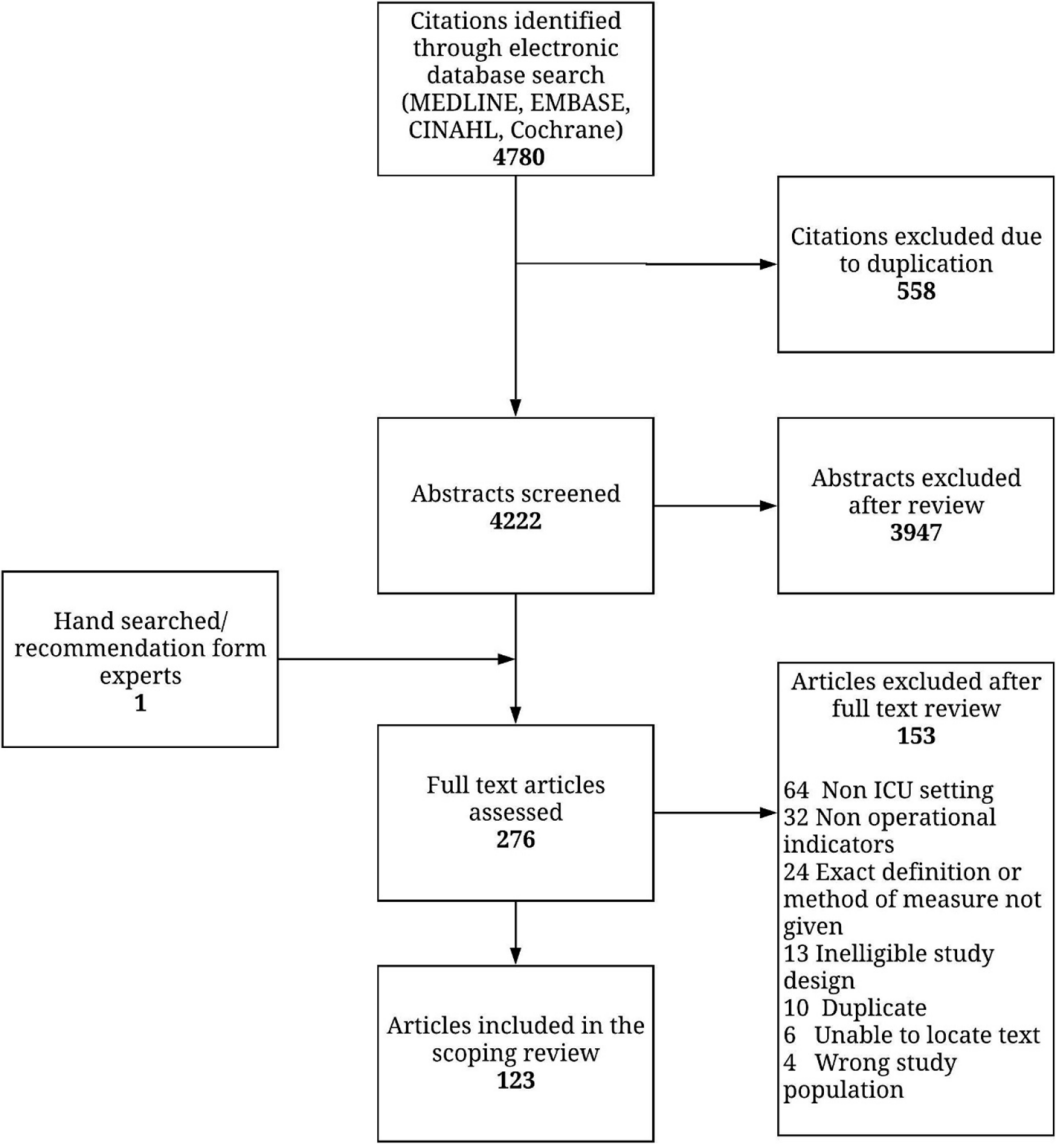
PRISMA flowchart. PRISMA flowchart summarizing study review and inclusion

The primary search of the literature yielded 4780 citations. Review of abstracts led to the retrieval of 276 full-text articles for assessment, of which 123 articles were retained for review [see Additional file 3]. The most common reason for excluding articles after full-text review was the absence of an operational QI (Fig. 1).

The majority of articles synthesized were original research (98.3%, 121), of which 88.6% (109) were cohort studies (Table 1).

**Table 1:** Literature characteristics

Type of article	*N* (%) total 123
Original research	
Cohort Study	109 (88.6%)
Cross sectional study	5 (4.1%)
Case series/ case report	0
Trial	2 (1.6%)
Other	5 (4.1%)
Non original research	
Reviews	1 (0.8%)
Other	1 (0.8%)
Reporting mechanism	
Near real time/ contemporaneous to delivery of care	112 (91.0%)
Retrospective	6 (4.8%)
Unknown (near real time/retrospective)	5 (4.0%)
Electronic	114 (92.7%)
Paper based	0
Both electronic and paper based	1 (0.8%)
Unknown	8 (6.5%)

### Characteristics of literature included in full review

**Fig. 2:**
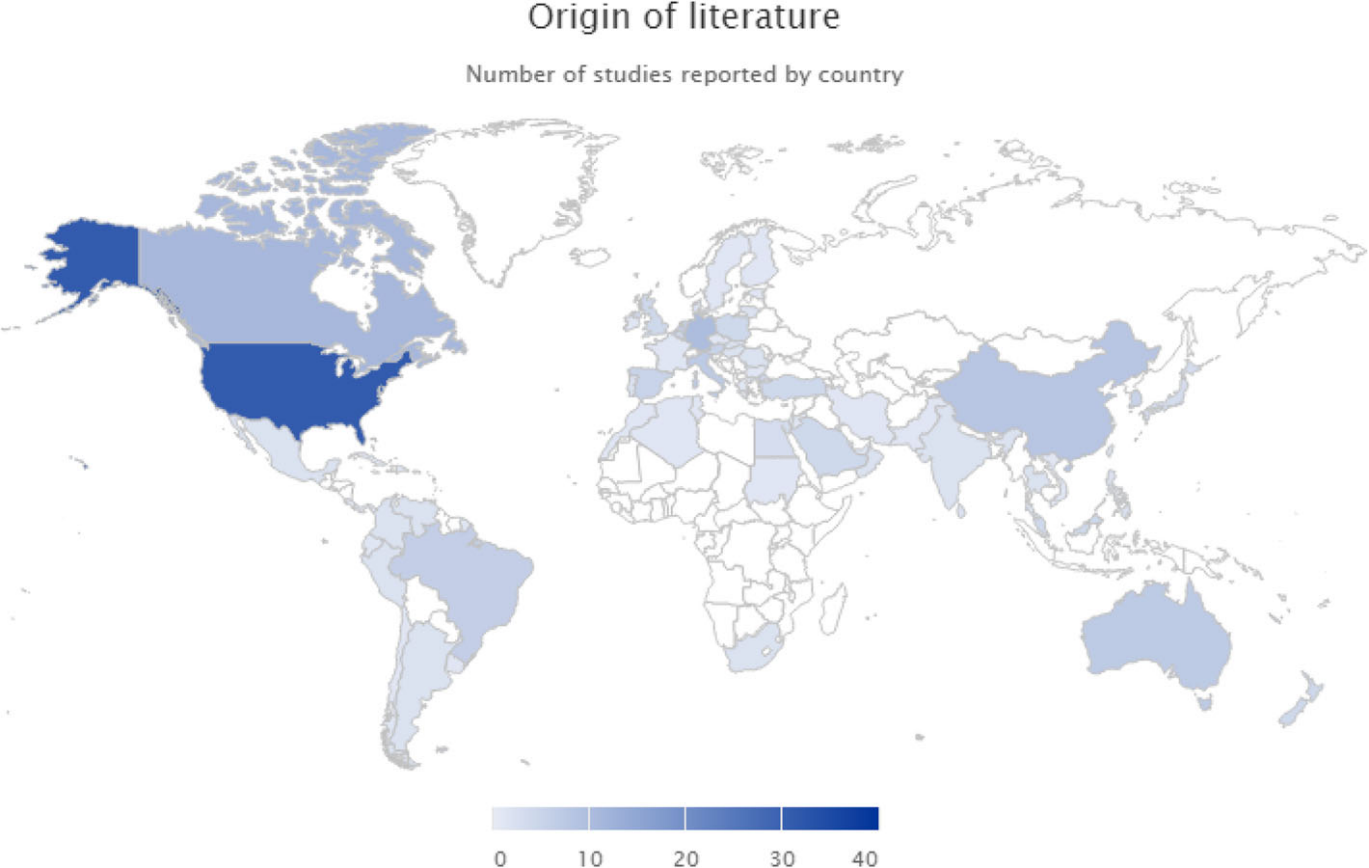
Geographical origin of literature. Origin of literature by country [using the UN Geoscheme classification—Accessed: at http://millenniumindicators.un.org/unsd/methods/m49/m49regin.htm**]**

The majority of the literature was reported from registries evaluating quality of critical care in high income country healthcare systems with 27.85% of research originating from Australia, Canada, Northern Europe, and the USA (Fig. 2).

### QIs, definition, measurement, and evidence base

From the 123 articles retained for full review, 253 indicators were identified, of which 51 were unique. These unique indicators were classified using prespecified components of the HQSS framework [13] and then by phase of ICU encounter (pre, in, and post ICU) (Table 2) [13].

**Table 2:** Results table of unique quality indicators

Category	Foundations (7)13.7%	Processes (28)54.9%	Quality impacts (16)31.3%
Pre ICU	–	Late unplanned ICU admission Early unplanned ICU admission Delayed ICU admission ICU referral burden	Mortality associated with weekend admission
In ICU	ICU occupancy ICU turnover Nursing time Intensivist staffing Patient to nurse ratio Nurse workload ICU night coverage	Stress ulcer prophylaxis Venous thromboembolism prophylaxis Duration of mechanical ventilation Incidence of ARDS Proportion of extubations which are re-intubated Nosocomial Resistance Index Compliance with antimicrobial guidance Empirical antibiotic therapy Density of antimicrobial use Incidence of bloodstream Infection Incidence of ventilator associated pneumonia Incidence of urinary catheter associated infection Incidence of central venous catheter associated infection Incidence of nosocomial infections Incidence of nosocomial MRSA ICU census accuracy ICU admission census ICU night discharge Transfer due to ICU capacity Avoidable days in ICU Patient flow Unplanned extubation Consent rate for solid organ donation	ICU mortality Risk adjusted mortality (Standardized Mortality Ratio). Predicted ICU mortality ICU length of stay Acuity adjusted length of stay, ICU readmission
Post ICU	–	Patient experience	Hospital mortality Predicted hospital mortality Relative risk mortality rate One year mortality Hospital length of stay Acuity-adjusted length of stay Weighted mean reduction in length of hospital stay Post ICU quality of life Psychological outcomes post ICU Patient satisfaction

Quality indicators identified by the scoping review classified by HQSS framework [13]

Foundational indicators accounted for 13.7% (7) of the 51 unique QIs identified, processes 54.9% (28), and quality impact 31.3% (16) respectively. Health care associated events (including healthcare associated infections) accounted for 37.1% (10) of process indicators and were present in 39.8% (49) of articles included in full text review. In contrast, patient reported outcome related measures accounted for less than 6% of quality impact indicators and were present in 1.6% (2) of articles reviewed.

The majority of QIs (58%) had a single definition and method of measurement. The mean number of different definitions for a single indicator was 1.61 (SD 0.72 to 2.50). Indicators with the most variation in definition were composite measures of mortality and adverse events, including hospital associated adverse events. Definitions varied based on inclusion or exclusion of laboratory tests, radiological imaging and constellations of clinical signs and symptoms, and varied depending on the country from which the underpinning evidence originated (Supplementary file 1). Of the 123 articles included in the full review, 96% (118) included a description of how the indicators were measured, and a further 88% (108) reported guidance on data collection (including frequency) and analysis.

### Barriers to implementation of quality indicators

Barriers to implementation were described in 35.7% of articles included in the full text review. The barriers identified broadly related to two aspects of implementation; “operational” (51%) (which included issues with data collection and data quality), and “acceptability and actionability” (49%) (which included users’ perceptions of the indicator, its validity, and the subsequent actionability of the information for care improvement) (Table 3).

**Table 3:** Barriers to implementation of quality indicators

Barriers identified	References
**Operational Barriers**	
• Concerns about inaccuracy associated with retrospective data entry • Time burden of data collection • Bias/ inability to reproduce/ verify data due to manual data collection • Poor replicability of data extraction process. • Data missingness	[29–51]
**Acceptability Barriers**	
• Indicator definition unactionable. • Required processes of care associated with indicator not measured limiting interpretability • Data quality concerns about self-reporting and responder biases • Concerns about the ramifications for individual clinician and ICU team performance	[35, 36, 40, 46, 52–71]

#### Operational

Reliance on manual data entry (as opposed to direct extraction from an electronic source-such as patient monitor or EHR) and the associated burden of data capture were reported impediments to operationalisation of the QIs in the ICU [36, 40, 44]. Absence of data (missingness) resulting in exclusion of patient encounters from analysis, was also described; inhibiting both implementation and utilisation of QIs. Missingness was described both as a consequence of manual data entry and as a result of unavailability of information at source [35, 39, 42, 52]. The impact of this missingness being that several studies had to exclude data in analysis [41, 43, 47, 48]. Challenges with data quality were described at point of data capture and extraction [29, 30, 32, 35, 38–40, 42, 46, 51, 54, 64, 72]. Ability to accurately measure processes of care associated with the indicator (e.g., time of antibiotic administration in the context of recognition of infection/ prescription of antibiotics) hindered utilisation of the indicator in care evaluations [33, 46, 53, 57, 58, 66, 67, 70, 71, 73].

#### Acceptability and actionability

Concerns about the ramifications for individual clinicians and ICU team performance was a barrier to implementing indicators of quality in ICU. Indicators relating to adverse events including hospital associated (nosocomial) infections and to measures of performance for which there may be significant public pressure (such as antimicrobial prescribing) were particularly associated with fear of blame [36]. These concerns impeded healthcare providers willingness to engage in the reporting of incidence and associated quality of care benchmarking within their department and a willingness to contribute data to interdepartmental and interhospital reporting (regional and national) [35–37, 56, 59, 68]. Similarly, it was identified as a contributing factor to missingness of data (described above) and a driver for revision of definitions [29, 52, 65]. These barriers were most evident in literature originating from North America and China.

Reliability and reproducibility of the data was a barrier to acceptability (and actionability) of the indicators. Concerns over information that was not captured electronically, but by direct observation from clinicians delivering care was considered at risk of responder biases [56, 60]. These concerns hindered researchers and clinicians’ willingness to accept findings, and in turn, impeded subsequent efforts to use the data to drive quality improvement initiatives in the clinical settings [36, 74]. Challenges in interpretability of indicators, which further limited acceptability were also identified. Indicators relating to ICU resource utilisation (occupancy, turnover and staff utilisation) were described as “difficult” to interpret given the dynamic nature of ICU service activity and patient acuity [46, 67]. Similarly, indicators pertaining to hospital associated infections (and other adverse events) were described as undergoing frequent revision of definition and therefore data capture methods, due to difficulties in interpreting the findings in the context of patient groups and care processes [29, 35, 52, 65]. As a consequence, these indicators were associated with frequent revision of definition and method of measurement. Interconnected, these revisions to definitions and data set further impeded quality of data collection and their acceptability of the findings for the healthcare team [35, 65].

### Impact of indicators on practice in the ICU

Of the literature reviewed 49.6% articles reported positive impacts on ICU practice following the reporting of quality indicators in the ICU. Measurement of process and quality impact indicators; infection control practice, and the incidence of hospital acquired infections were cited as being the catalyst for quality improvement interventions [30, 31, 35, 40, 61, 65, 82, 87, 99, 119, 134]. Interventions described included development of guidelines, the establishment of an infection control team and the use of screening tools for patients at risk of avoidable infections. However there was limited explanation of how the indicator resulted in identification of modifiable care processes, or if the subsequently reported reduction in incidence of adverse events resulted in improvement in patient outcomes.

A positive impact associated with the measurement of indicators was an observed shift in organizational culture toward greater awareness of care quality. The process of establishing routine measurement of indicators of quality in the ICU was perceived to contribute to an increased awareness of quality among healthcare team members and greater attention from staff regarding adherence to existing practice guidelines. This positive impact was most notable when clinical teams used the indicators as part of an audit and feedback initiatives, and as a target to drive forward actionable change as part of the wider quality improvement cycle.

## Discussion

Given the complexity, intensity, and risks of care in ICU, this review of published literature revealed a limited number of indicators in operation internationally. Despite critical care being an increasingly central tenet of healthcare service provision internationally, both the origin of the literature describing these indicators, and the evidence on which they are founded was concentrated from high-income country health systems—most notably North America, Canada, and China. Unusually for health systems globally, the USA and Canada notably have well-established EHRs. Such infrastructure is not representational of the majority of health systems, especially in resource constrained settings, where access to EHRs are uncommon. Absence of investment in such infrastructure is well described in low-and middle-income countries (LMICS) as a barrier to both operationalizing quality indicators in ICUs internationally, and as a contributor to the lack of published literature evaluating quality of critical care services [13, 15].

The majority of indicators identified described HQSS measures of care processes and quality impacts [13]. These indicators are composite, meaning that measurement of the indicator requires more than one type of data, and data to be captured at more than one time point [10]. Example composite indicators included device-associated bloodstream infection, antimicrobial resistance, and ventilation-associated pneumonia. Consequently, implementing such indicators can be problematic due to the burden of data capture they necessitate. The density and volume of information needed to accurately determine such indicators (which in the example of hospital-acquired infections (HAIs) may include multiple timepoints in every 24 h) may be especially troublesome to implement in resource constrained settings, where data capture is likely to by hand, drawing information from multiple sources. The wide variation and iteration in both definition and measurement of indicators described in the literature highlights perhaps how stakeholders have attempted to overcome the challenge of data capture and interpretation. Iterations to definitions include attempts to reduce complexity of measure, reduce the burden of data capture, or remove dependency on diagnostic markers to simplify definitions used to determine incidence [29].

Whilst commonly used to benchmark care, it is widely accepted that such composite measures are difficult to elucidate, not least because their interpretation is complicated by both complexity of disease and care processes within ICU. Moreover, they are both subject to underreporting and difficult to risk adjust, further complicating their utility for benchmarking. Tending to focus on the presence of an adverse event or omission in care processes, they are used to infer that quality of care may be suboptimal. However in focusing on the negative, these indicators and their measurement provides little insight for teams seeking to inform actionable improvement or reinforce good practice. This reinforces the perception that quality indicators are used as a weapon to criticise care delivery. Described as a barrier to operationalising QIs, stakeholders questioned the validity of the indicator and failed to engage in using them to guide practice improvement. Very few of the indicators focus on the presence or inclusion of actions which contribute to positive care outcomes, and for which definition, measurement and interpretation may be arguably more acceptable to the clinical team. Notable exceptions were, indicators focusing on compliance with antimicrobial guidelines, and administration of therapies to prevent adverse events associated with critical illness including anticoagulation for Venous Thromboembolism (VT) prevention and gastric ulcer prophylaxis. It was these indicators that were most closely associated with positive change in the ICU. Particularly it was their use as part of a cycle of audit and feedback to drive improvement that was perceived to have a positive impact on care [75]. Increasing research to select and develop indicators includes assessment of their ability to lead to actionable improvement, in addition to their prognostic validity, feasibility and applicability of capture. The repeated adjustment to definition or measurement of composite indicators described in literature may well be a further illustration of healthcare providers (and researchers) growing uncomfortableness and distrust of such indicators being used to measure clinical performance [29, 35, 65].

Despite a growing acknowledgment of the need to better understand the organizational, social, and economic impact of and recovery following critical illness, very few articles described the operationalisation of QIs reporting the quality of recovery following ICU care or patient-centered measures of experience, suggesting a need for further empirical research in these aspects of care.

### Limitations

There are limitations to this review. Despite the search of multiple databases using comprehensive search strategies with the assistance of experts in critical care registries, it is likely that our search missed broad categories of important QIs. This is in part due to the heterogeneity of language used to describe the indicators. In addition, it was difficult to extract accurate data from all publications. Two percent of articles were unavailable for full text and some did not disclose the materials or methods used, and in 9% of articles the exact mechanism of capture or definition was not described. Finally, despite categorizing articles using predefined data abstraction tools and classification schemes, classification remains subjective. To minimize this, a third reviewer reviewed 10% of articles independently to verify consistency of categorization.

## Conclusion

This scoping review has evaluated the growing body of literature on the implementation of QIs in critical care settings and found that, despite the complexity and risk associated with ICU care, there are only a small number of operational indicators used. These mostly focus on processes of care, especially healthcare-associated adverse events. These predominantly composite measures requiring multiple data points captured at more than one time, are associated with a high burden of data capture and are difficult to evaluate in the context of heterogeneous patient populations and diverse health systems.

Similarly, the majority of literature (and evidence underpinning the definition of indicators) originates from high-income country health systems, and are not necessarily representational of the case mix, care processes, or patient-centered priorities for recovery from low- and middle-income countries. Future selection of QIs would benefit from a stakeholder-driven approach, whereby the values of patients and communities and the priorities for actionable improvement as perceived by healthcare providers are prioritized. Such an approach may go some way to address not only the paucity of patient centered outcome measures in operation but also the barriers of acceptability and actionability identified by this review. In doing so reduce the observations of fear and blame associated with QIs used to benchmark care and drive improvement.

## Supplementary Information

**Additional file 1.** PRISMA-ScR Checklist.

**Additional file 2.** Supplementary File 1: Definitions of unique quality indicators and evidence grading [29–50, 52–69, 72–74, 76–145].

**Additional file 3.** Supplementary File 2: List of articles included in the review.

## Data Availability

Not applicable.

## Abbreviations

CDC-NHSN: Center for Disease Control - National Healthcare Safety Network
CFU: Colony-forming unit
CPIS: Clinical Pulmonary Infection Score
EHR: Electronic Health Record
HAIs: Hospital acquired infections
HELICS: Hospitals in Europe Link for Infection Control through Surveillance
HQSS: High Quality Health Systems
ICED: Intensive Care Experience Definition
ICNARC: Intensive Care National Audit And Research Centre
ICU: Intensive care unit
KONIS: Korean Nosocomial Infections Surveillance System
LMICS: Low- and middle-income countries
NICE: National Institute for Clinical Excellence
PEEP: Positive end expiratory pressure
QI: Quality indicator
QoL: Quality of life
SMR: Standardized mortality ratio
VT: Venous thromboembolism
WHO: World Health Organisation
