# Supplementary material for: A scoping review of registry captured indicators for evaluating quality of critical care in ICU
Source: J Intensive Care. 2021 Aug 5;9:48. doi: 10.1186/s40560-021-00556-6 (PMC8339165; doi:10.1186/s40560-021-00556-6)
Supplement: Supplementary file 2 — Additional file 2. Supplementary File 1: Definitions of unique quality indicators and evidence grading [29–50, 52–69, 72–74, 76–145]. [file 40560_2021_556_MOESM2_ESM.docx]

**Supplementary File 1:** Definitions of unique quality indicators and evidence grading.

| **Quality indicator** | **No. of studies [Reference]** | **Definitions** | **Level of qualifying evidence** | **Grade of evidence** |  |
| --- | --- | --- | --- | --- | --- |
| **Foundation** | | | | |  |
| ICU night coverage | 1 [29]  1 [30]  1 [31] | Definition 1: IMPACT: Unknown  Definition 2: Proportion of patients discharged to the ward between 10 pm and 8 am (out-of-hours)  Definition 3:  Number of patients discharged alive to a ward, step-down, high-dependency, high observation, or another non-ICU patient area in the same hospital, between the hours of 22:00 and 06:59, calculated as a percent of all live ICU discharges. | Levels II, III, or IV evidence and findings are generally consistent | B |  |
|  |  |  |  |  |  |
|  |  |  |  |  |  |
| Nursing time | 1 [32] | Definition: Mean time of nursing care, per patient, spent by the professional category k, at duty | Level I evidence or consistent findings from multiple studies of levels II, III, or IV | A |  |
| Intensivist staffing | 1 [33]  1 [34] | Definition 1: The highest level of intensivist involvement by day 4 of mechanical ventilation at the patient level  Definition 2: Assessed if the patient was cared for by an intensivist during any period of their admission | Levels II, III, or IV evidence and findings are generally consistent | B |  |
|  |  |  |  |  |  |
| Patient to nurse ratio | 1 [35]  1 [36] | Definition 1: Number of patients assigned to one nurse  Definition 2: Patient-to-nurse ratio per shift = Total inpatient days / (Number of adult ICU nurse staffing ∗ 1800 h / 24 h), where the total inpatient days were the sum of inpatient days in the adult ICU per year, and 1800 h represented the worked hours per nurse per year | Level I evidence or consistent findings from multiple studies of levels II, III, or IV | A |  |
|  |  |  |  |  |  |
| Nurse workload | 1 [37] | Definition: Nurse workload, as assessed by the Nursing Activities Score (NAS) | Levels II, III, or IV evidence and findings are generally consistent | B |  |
| **Processes of care** | | | | |  |
| Nosocomial Resistance Index | 1 [38] | Definition: NRI = Numerator is the number of nosocomial isolates (or, in the case of Clostridium difﬁcile, the number of toxin-positive specimens) of 6 different organisms: (1) methicillin-resistant Staphylococcus aureus (MRSA), (2) vancomycin-resistant Enterococcus, (3) C. difﬁcile, (4) ﬂuoroquinolone-resistant Pseudomonas aeruginosa, (5) ceftazidime-resistant gram-negative bacilli, and (6) Stenotrophomonas maltophilia. Only isolates that are recovered more than 48 hours after hospital admission are included. Antibiotic usage is defined by WHO daily drug doses. | Levels II, III, or IV evidence, but findings are inconsistent | C |  |
| Compliance with antimicrobial guidance | 1 [39] | Definition: Antibiotics started 48 hours after admission were cross referenced with Veteran Health Association antibiotic guidelines and any which were off protocol were deemed to be non-compliant. | Levels II, III, or IV evidence and findings are generally consistent | B |  |
| Empirical antibiotic therapy | 1 [40] | Definition: On their automated registry, an antibiotic indication must be entered. Empiric antibiotic therapy was considered in the two following scenarios: (1) An infection is suspected, but the infection type is unknown, and treatment is therefore ‘‘empiric’’; or (2) a certain type of infection is suspected, but the organism(s) remains unknown (e.g., ‘‘empiric–pneumonia’’).  Targeted treatment was defined when antibiotics were prescribed for patients meeting the CDC definitions of site-specific infections. | Level I evidence or consistent findings from multiple studies of levels II, III, or IV | A |  |
| Density of antimicrobial use | 2 [41, 42] | Definition: Total antibiotic consumption in grams was divided into Defined Daily Dose as per WHO and then reported as Defined Dose per 1000 patient-days | Levels II, III, or IV evidence and findings are generally consistent | B |  |
| Stress ulcer prophylaxis | 1 [43] | Definition: Use of a histamine-2 receptor blocker or proton pump inhibitor  In any patients receiving mechanical ventilation for at least 48 of 60 hr | Levels II, III, or IV evidence and findings are generally consistent | B |  |
| Venous thromboembolism prophylaxis | 2 [43, 44] | Definition: Mechanical or pharmacologic prophylaxis in all patients without bleeding admission diagnoses or complications. | Levels II, III, or IV evidence and findings are generally consistent | B |  |
| Unplanned extubations | 1 [31]  1 [45] | Definition 1: Unplanned extubation is the unscheduled removal of an artificial airway (endotracheal or tracheostomy tube) due to accidental dislodgement or patient self extubation. The patient need not be ventilated at the exact time of the event (e.g., on t-piece or tracheal mask).  Definition 2: The primary outcomes were the percentage of extubated patients who were reintubated and the cumulative probability of reintubation, accounting for the competing risks of death and/or placement of a DNR order. Time of extubation was defined using the end date and time of invasive mechanical ventilation. Reintubation was defined by the reinstitution of invasive mechanical ventilation following extubation at any time during the same ICU stay. | Levels II, III, or IV evidence, but findings are inconsistent | C |  |
|  |  |  |  |  |  |
| Duration of mechanical ventilation | 1 [46] | Definition: Duration defined as number of days on mechanical ventilation in ICU. Prolonged ventilation defined as mechanical ventilation for greater than 21 days | Level I evidence or consistent findings from multiple studies of levels II, III, or IV | A |  |
| Incidence of ARDS | 1 [47] | Definition: Diagnosis of ARDS was based on the Berlin definition for ARDS: Inset over one week or less - Bilateral opacities consistent with pulmonary oedema on computed tomography scan or chest radiograph - PF ratio ,300 mm Hg with a minimum of 5 cm H2O positive end-expiratory pressure (PEEP), or continuous positive airway pressure (CPAP) - Above conditions are not fully explained by cardiac failure or fluid overload | Levels II, III, or IV evidence and findings are generally consistent | B |  |
| Incidence of Nosocomial bloodstream Infection | 7 [48, 49, 50, 51, 52, 53, 54]  6 [55, 56,57, 58, 59, 60]    3 [61, 62, 63] | Definition 1 - CDC-NHSN: Recognized pathogen cultured from one or more blood cultures and organism cultured from blood is not related to an infection at another site  OR  Fever (> 38 °C), chills, or hypotension and common commensal is cultured from two or more blood cultures drawn on separate occasions.  Definition 2 -HELICS: One positive blood culture for a recognised pathogen.  OR  One of the following signs or symptoms: fever (> 38 °C), chills, or hypotension and two positive blood cultures for a common skin contaminant (from two separate blood samples, usually within 48 hours).  Definition 3 - Chinese Ministry of Health: Definition not available in English | Level I evidence or consistent findings from multiple studies of levels II, III, or IV | A |  |
|  |  |  |  |  |  |
|  |  |  |  |  |  |
| Incidence of Ventilator Associated Pneumonia | 18 [48, 49, 50, 53, 64, 65, 66, 67, 68, 69, 70, 71, 72, 73, 74, 75, 76, 77]  4 [55, 56, 57, 78]  3 [61, 62, 63]  1 [64] | Definition 1 - CDC-NHSN: Patient has a baseline period of ≥2 calendar days of stable or decreasing daily minimum FiO2 or PEEP values. The patient then has at least one of the following indicators of worsening oxygenation:    1. Increase in daily minimum FiO2 of ≥0.20 (20 points) over the daily minimum FiO2 in the baseline period, sustained for ≥2 calendar days.  2. Increase in daily minimum PEEP values of ≥3 cmH2O over the daily minimum PEEP in the baseline period, sustained for ≥2 calendar days.  AND   Temperature >38°C or <36°C, or white blood cell count ≥12,000 cells/mm3 or ≤4,000 cells/mm3  AND   A new antimicrobial agent(s)) is started and is continued for ≥4 calendar days.  AND  ONE of the following criteria is met:  1. Purulent respiratory secretions (from one or more specimen collections): a. Defined as secretions from the lungs, bronchi, or trachea that contain ≥25 neutrophils and ≤10 squamous epithelial cells per low power field [lpf, x100].   b. If the laboratory reports semi-quantitative results, those results must be equivalent to the above quantitative thresholds.  2. Positive culture (qualitative, semi-quantitative or quantitative) of sputum, endotracheal aspirate, bronchoalveolar lavage, lung tissue, or protected specimen brushing (Excludes the following: • Normal respiratory/oral flora, mixed respiratory/oral flora or equivalent • Candida species or yeast not otherwise specified • Coagulase-negative Staphylococcus species • Enterococcus species).  Definition 2 - HELICS: Two or more serial chest X-rays/ CT-scans suggesting pneumonia in patients with underlying cardiac/respiratory disease or one if no underlying disease.  AND  Symptoms: fever > 38 °C, leukopenia (< 4 000 WBC/mm3) or leucocytosis (≥ 12 000 WBC/mm3).  AND  One of the following if microbiology results present OR two if no microbiology:  New onset of purulent sputum, or change in character of sputum (colour, odour, quantity, consistency) cough or dyspnoea or tachypnoea suggestive auscultation (rales or bronchial breath sounds), rhonchi, wheezing worsening gas exchange (e.g., O2 desaturation or increased oxygen requirements or increased ventilation demand).  AND (if only one clinical symptom present):  Positive quantitative culture from minimally contaminated LRT specimen:  Broncho-alveolar lavage (BAL) with a threshold of ≥ 104 colony forming units (CFU)/ml or ≥ 5% of BAL-obtained cells contain intracellular bacteria on direct microscopic exam.  Protected brushings with a threshold of ≥ 103 CFU/ml  distal protected aspirate (DPA) with a threshold of ≥ 103 CFU/ml.  Positive quantitative culture from possibly contaminated LRT specimen with a threshold of 106 CFU/ml.  Positive blood culture not related to another source of infection.  Positive growth in culture of pleural fluid, pleural or pulmonary abscess with positive needle aspiration or histologic pulmonary exam shows evidence of pneumonia.  Positive exams for pneumonia with virus or particular germs (Legionella, Aspergillus, mycobacteria, mycoplasma, Pneumocystis carinii):  positive detection of viral antigen or antibody from respiratory secretions (e.g. EIA, FAMA, shell vial assay, PCR) positive direct exam or positive culture from bronchial secretions or tissue seroconversion (example: influenza viruses, Legionella, Chlamydia) detection of antigens in urine (Legionella).  Definition 3 - Chinese Ministry of Health: Definition not available in English  Definition 4 - CPIS:  Total >6 is VAP:  Body temperature: ≥ 36.5 or ≤ 38.4 = 0 point ≥ 38.5 or ≤ 38.9 = 1 point ≥ 39 or < 36.5 = 2 point  Pulmonary infiltration in chest X-ray: No infiltration = 0 point Diffuse infiltration = 1 point Localized infiltration = 1 points  Leukocyte count: microscopy ≥ 4000 or ≤ 11.000 = 0 point < 4000 or > 11.000 = 1 point Rod form ≥ % 50 = Add 1 point  Progression in pulmonary infiltration: Radiographic progression (-) = 0 point Radiographic progression (+) (After the exclusion of HF and ARDS) = 2 points  Tracheal secretion: Tracheal secretion (-) = 0 point Tracheal secretion with less purulence = 1 point Abundant purulent secretion = 2 points  Pathogenic bacteria in tracheal aspirate culture:  No or few pathogenic bacteria = 0 point Moderate or high levels of pathogenic bacteria = 1 point Pathogenic bacteria to be seen in Gram staining, add 1 point  Oxygenation: Pa02/Fi02, mmHg > 240 or ARDS (ARDS: Pa02/Fi02 < 200, Pa02/Fi02 < 200, PAWP ≤ 18 mmHg and bilateral acute infiltration) = 0 point Pa02/Fi02, mmHg ≤ 240 or ARDS = 2 points | Level I evidence or consistent findings from multiple studies of levels II, III, or IV | A |  |
|  |  |  |  |  |  |
|  |  |  |  |  |  |
|  |  |  |  |  |  |
| Incidence of Urinary Catheter associated Infection | 11 [48, 49, 53 68, 69, 70, 73, 74, 76, 79, 80]  2 [55, 57]  3 [61, 62, 78] | Definition 1 - CDC-NHSN:  48 hours after insertion of indwelling urinary catheter or less than 24 hours after removal  AND  Fever (>38°C), urgency, frequency, dysuria, suprapubic tenderness, costovertebral angle pain or tenderness  AND  Positive urine culture of ≥105 colony-forming units (CFU)/ml with no more than 2 species of microorganisms.  Definition 2 - HELICS:  48 hours after insertion of indwelling urinary catheter or less than 48 hours after removal  AND  One of the following symptoms with no other recognised cause: fever (> 38 °C), urgency, frequency, dysuria, or suprapubic tenderness  AND  Patient has a positive urine culture, i.e. ≥ 105 microorganisms per ml of urine with no more than two species of microorganisms.  OR  One of the following:  -Positive dipstick for leukocyte esterase and/or nitrate  -Pyuria urine specimen with ≥ 10 WBC/ml or ≥ 3 WBC/high-power field of unspun urine organisms seen on Gram stain of unspun urine.  - At least two urine cultures with repeated isolation of the same uropathogen ≤ 105 colonies/ml of a single uropathogen (Gram-negative bacteria or S. saprophyticus) in a patient being treated with effective antimicrobial agent for a urinary infection physician diagnosis of a urinary tract infection physician institutes appropriate therapy for a urinary infection.  Definition 3 - Chinese Ministry of Health: Definition not available in English | Level I evidence or consistent findings from multiple studies of levels II, III, or IV | A |  |
|  |  |  |  |  |  |
|  |  |  |  |  |  |
| Incidence of central venous catheter associated infection | 16 [44, 48, 49, 50, 53, 68, 69, 70, 73, 74, 76, 81, 82, 83, 84, 85]  7 [55, 56, 57, 82, 86, 87, 88]    3 [61, 62, 63] | Definition 1 - CDC-NHSN: Eligible central venous catheter (CVC) (48hours after insertion and <48 hours from removal)  AND  Recognized pathogen cultured from one or more blood cultures and organism cultured from blood is not related to an infection at another site  OR  Fever (> 38 °C), chills, or hypotension and common commensal is cultured from two or more blood cultures drawn on separate occasions.  Definition 2 - HELICS:  Eligible central venous catheter (CVC) (48hours after insertion and <48 hours from removal)  AND  Quantitative CVC culture (103 CFU/ml) or semi-quantitative CVC culture (>15 CFU) and pus/inflammation at the insertion site or tunnel or clinical signs improve within 48 hours after catheter removal.  OR  Bloodstream infection (as defined earlier) and Positive culture with the same microorganism of either:  -Quantitative CVC culture ≥ 103 CFU/ml or semi-quantitative CVC culture > 15 CFU  -Quantitative blood culture ratio: CVC blood sample/peripheral blood sample > 5 differential delay of positivity of blood cultures  -CVC blood sample culture positive two hours or more before peripheral blood culture (blood samples drawn at the same time)  -positive culture with the same microorganism from pus from insertion site.  Definition 3 - Chinese Ministry of Health: Definition not available in English | Level I evidence or consistent findings from multiple studies of levels II, III, or IV | A |  |
|  |  |  |  |  |  |
|  |  |  |  |  |  |
| Incidence of Nosocomial infections (not device specific - aggregate measure reported) | 6 [53, 89, 90, 91, 92, 93]  3 [57, 94, 95]  2 [63, 96] | Definition 1 - CDC-NHSN: as above  Definition 2 - HELICS: as above  Definition 3 - Chinese Ministry of Health: Definition not available in English | Level I evidence or consistent findings from multiple studies of levels II, III, or IV | A |  |
|  |  |  |  |  |  |
|  |  |  |  |  |  |
| Incidence of nosocomial MRSA | 4 [97, 98, 99, 100] | Definition - HELICS: Any microbiological sample which is positive for MRSA regardless of source, in a patient who does not have MRSA on admission (defined as being colonized or infected by MRSA before or not later than 48 hours after ICU admission). | Levels II, III, or IV evidence and findings are generally consistent | B |  |
| ICU occupancy | 2 [35, 101]  1 [102]  1 [31]  1 [103] | Definition 1: Percentage of ICU bed capacity occupied at the time of ICU referral, admission, or discharge  Definition 2: Number of patients using the ICU in a given 24-hour period for at least 2 hours, standardized for each ICU and year  Definition 3: Average occupancy is calculated as the sum of the average maximum census and average minimum census divided by twice the number of ICU beds. An ICU bed is defined as the number of beds regularly available for patient care, regardless of staffing. Occupancy is expressed as percent.  Definition 4: Percentage of beds occupied or reserved for surgical patients | Levels II, III, or IV evidence and findings are generally consistent | B |  |
|  |  |  |  |  |  |
|  |  |  |  |  |  |
|  |  |  |  |  |  |
| ICU turnover | 1 [101] | Definition: Percentage of ICU bed capacity newly admitted to the ICU during the 24 hours after ICU admission, the 24 hours before ICU discharge, or the calendar day of ICU referral | Levels II, III, or IV evidence and findings are generally consistent | B |  |
| ICU census acuity | 2 [ 44, 101]  1 [102] | Definition 1: Mean predicted hospital mortality of all patients admitted to the ICU (excluding the index patient) at the time of ICU referral, admission, or discharge. Predicted hospital mortality was calculated by the Mortality Probability Admission Model-III (MPM0-III),16 a composite model based on clinical and historical data obtained within 1 hour of ICU admission. For patients with missing MPM0-III-predicted hospital mortalities, we used the mean MPM0-III-predicted hospital mortalities from 10 imputed data sets (see Appendices 2 and 3 for details).  Definition 2: Average predicted probability of death of the other patients in the ICU that day | Levels II, III, or IV evidence and findings are generally consistent | B |  |
|  |  |  |  |  |  |
| ICU referral burden | 1 [101] | Definition: Number of patients, expressed as a percentage of ICU bed capacity, who were referred but declined for ICU admission on the calendar day of ICU admission, discharge, or referral. | Levels II, III, or IV evidence and findings are generally consistent | B |  |
| ICU admissions | 1 [102]  1 [44] | Definition 1: Proportion of patients who were new admissions divided by the total number of patients cared for at least 2 hours in that ICU on that day  Definition 2: The proportion of new admissions was calculated as the proportion of the total census that had been admitted on a given day | Levels II, III, or IV evidence and findings are generally consistent | B |  |
|  |  |  |  |  |  |
| ICU readmission | 3 [29, 43 102]  2 [30, 104]  1 [31] | Definition 1: ICU readmission was defined using increasing durations of time between ICU discharge and readmission in 6-hour increments from 24 to 168 hours (1–7 days). Each increment included cumulative readmission counts (e.g., the 36- h definition included all 30-h readmissions plus those occurring between 30 and 36 h). We chose a lower limit of 24 hours because few readmissions occurred before that, and an upper limit of 168 hours because readmissions after that point are unlikely to be because of ICU care  Definition 2: Proportion of patients readmitted to ICU within 48 hours of discharge  Definition 3: Number of patients with an unplanned readmission to ICU within 72 hours of ICU discharge within the same hospitalization, calculated as a percent of live discharges | Levels II, III, or IV evidence and findings are generally consistent | B |  |
|  |  |  |  |  |  |
|  |  |  |  |  |  |
| Transfer due to ICU capacity | 1 [30]  1 [103] | Definition 1: Proportion of patients transferred to another ICU for capacity reasons  Definition 2: Critically ill patients not admitted due to a lack of beds in the ICU in the last 24 h | Levels II, III, or IV evidence and findings are generally consistent | B |  |
|  |  |  |  |  |  |
| Avoidable days in ICU | 1 [31] | Definition:  The amount of time that a patient occupies an ICU bed when ICU care is no longer required. The amount of time that patients occupy an ICU bed for more than 4 hours after a transfer order is written is considered avoidable. Avoidable days (24 hours) are expressed as a percent of total patient days. | Levels II, III, or IV evidence, but findings are inconsistent | C |  |
| Patient flow | 1 [31] | Definition: Number of patients per bed per year | Levels II, III, or IV evidence, but findings are inconsistent | C |  |
| Late unplanned readmission | 1 [105] | Definition: Incidence of first unplanned late rehospitalization (occurring between 31 and 180 days of hospital discharge) for patients surviving 30 days after discharge without death or rehospitalization | Levels II, III, or IV evidence and findings are generally consistent | B |  |
| Early unplanned ICU admission | 1 [105]  1 [37] | Definition 1: Unplanned readmissions from 24 hours – 30 days from discharge from hospital in patients who had been in ICU.  Definition 2: Early unplanned ICU readmissions (<24 hours and 48 hours after discharge) | Levels II, III, or IV evidence and findings are generally consistent | B |  |
|  |  |  |  |  |  |
| Quality impacts | | | | |  |
| ICU mortality  crude | 1 [101]  2 [29, 106]  1 [86]  1 [107]  15 [37, 56, 58, 77, 89, 108, 109, 110, 111, 112, 113, 114,115, 116, 117] | Definition 1 - ICED: The ICU mortality was defined as a death in the ICU or a palliative discharge from the ICU.  Definition 2 - IMPACT: ICU mortality included patients dying during the first ICU admission  Definition 3 - Danish Intensive Care Database: Death in the ICU  Definition 4 - ICNARC: ICU mortality - Excluding readmissions to the critical care unit during the hospital stay  Definition 5- ICU mortality controlling for significant covariates | Level I evidence or consistent findings from multiple studies of levels II, III, or IV | A |  |
|  |  |  |  |  |  |
|  |  |  |  |  |  |
|  |  |  |  |  |  |
|  |  |  |  |  |  |
|  |  |  |  |  |  |
| ICU - Standardized Mortality Ratio | 1 [30]  2 [118, 119]  10 [37, 120, 121,122, 123, 124, 125, 126, 127, 128] | Definition 1 - Danish Intensive Care Database: SMR is computed by indirect standardization as the ratio between the observed number of deaths and the expected number of deaths for the case-mix of each ICU.  Definition 2 - Critical Care Data System: The SMR for each ICU was calculated by dividing the observed mortality rate by the expected mortality rate for that ICU. The expected mortality rate was calculated by taking the average of the predicted mortality rates for all the patients from a specific ICU. The observed mortality rate was derived by counting the number of deaths and dividing by the total number of patients.  Definition 3 - NICE: The ratio of observed to expected hospital deaths. | Levels II, III, or IV evidence and findings are generally consistent | B |  |
|  |  |  |  |  |  |
|  |  |  |  |  |  |
|  |  |  |  |  |  |
| Hospital mortality | 9 [37, 77, 116, 117, 129, 130, 131,132, 133] | Definition: Dead or alive at hospital discharge | Level I evidence or consistent findings from multiple studies of levels II, III, or IV | A |  |
| Predicted hospital mortality | 1 [101] | Definition - ICED: Mean predicted hospital mortality of all patients admitted to the ICU. Predicted hospital mortality was calculated by the Mortality Probability Admission Model-III (MPM0-III),16 a composite model based on clinical and historical data obtained within 1 hour of ICU admission. | Levels II, III, or IV evidence and findings are generally consistent | B |  |
| Post ICU quality of life | 1 [134] | Definition: The SF-36 was used to assess health-related QOL. For each variable item, scores are coded, summed, and transformed on to a scale from 0 (worst possible health state measured by the questionnaire) to 100 (best possible health state) (Ware & Sherbourne, 1992).  Within each domain, a score of <50 = “low value of QOL” and >50 “high”. | Levels II, III, or IV evidence and findings are generally consistent | B |  |
| Psychological outcomes post ICU | 1 [134] | Definition: Anxiety and Depression diagnosed with a HADS score of >10.  PTSD measured using the Impact of Event Scale—Revised (IES-R). Score of >20 indicates reactions of clinical importance and a score > 35 indicates a high probability of post-traumatic stress disorder. | Levels II, III, or IV evidence and findings are generally consistent | B |  |
| ICU length of stay | 1 [50]  18 [58, 74, 77, 100, 104, 106, 110,113, 114, 116, 123, 127, 128, 133, 136, 137, 138, 139] | Definition 1 - KONIS:  The average length of ICU stay was defined as the calculated sum of stays of ICU patients aged >15 years, divided by patient day.  Definition 2: ICU length of stay was calculated using the exact interval (measured in minutes) between the day and time of ICU admission and the day and time of ICU discharge. Exact ICU length of stay was expressed as days and fractions of days. | Levels II, III, or IV evidence and findings are generally consistent | B |  |
|  |  |  |  |  |  |
|  |  |  |  |  |  |
| Hospital length of stay | 5 [37, 77, 116, 117, 135] | Definition; Index hospital admission date to hospital discharge - days. | Level I evidence or consistent findings from multiple studies of levels II, III, or IV | A |  |
| Acuity adjusted hospital length of stay | 2 [121, 130] | Definition:  Index hospital admission date to hospital discharge - days.  Length of Stay (LoS) adjusted for severity of illness (APACHE IV) | Level I evidence or consistent findings from multiple studies of levels II, III, or IV | A |  |
| Acuity adjusted ICU length of stay | 2 [121, 130] | Definition: Index ICU admission date to ICU discharge - days.  LoS adjusted for severity of illness (APACHE IV) | Level I evidence or consistent findings from multiple studies of levels II, III, or IV | A |  |
| Relative risk mortality rate | 1 [140] | Definition: not defined | Level V evidence: little or no systematic empirical evidence | D |  |
| Weighted mean reduction in length of hospital stays | 1 [140] | Definition: not defined | Level V evidence: little or no systematic empirical evidence | D |  |
| Patient satisfaction and experience | 1 [141] | ICU experiences, stress and satisfaction were gathered using a telephone questionnaire adapted from two previous studies. Questions related to satisfaction with ICU care, stressful experiences, memories retained by the patient, the ICU environment, interaction with healthcare professionals, dreams, nightmares, sleep disturbances and difficulties in concentrating were included. Impact on quality of life for patients’ families and the associated burden of having a loved one in critical care were not explored. Questions pertaining to direct experience of aspects of care were measured on a five-point Likert scale, as proposed in the original studies; where 0 = ‘I don’t remember’, 1 = ‘It was not hard’, 2 = ‘It was indifferent’, 3 = ‘It was hard’, 4 = ‘It was very hard’ and 5 = ‘It was awful’. Patient satisfaction with the different aspects of care were also measured on a Likert scale where 1 = Excellent, 2 = Very good, 3 = Good, 4 = Fair, 5 = Poor and 6 = Not Applicable | Levels II, III, or IV evidence and findings are generally consistent | B |  |
| Mortality associated with weekend admission | 1 [142] | Definition: SMR as adjusted for weekday and weekend admission. Weekend admission as any admission to the ICU occurring between Friday 19:00 and Monday 07:00 | Level I evidence or consistent findings from multiple studies of levels II, III, or IV | A |  |

Results table of identified unique quality indicators with their definition, measurement and evidence base classified using the Evidence-based clinical practice guidelines published by American Society of Plastic Surgeons, Burns PB, Rohrich RJ, Chung KC and reproduced here - Level 1: High-quality, multi-centered or single-centered, randomized controlled trial with adequate power; or systematic review of these studies, Level 2: Lesser-quality, randomized controlled trial; prospective cohort study; or systematic review of these studies, Level 3: Retrospective comparative study; case-control study; or systematic review of these studies, Level 4: Case series, Level 5: Expert opinion; case report or clinical example; or evidence based on physiology, bench research or “first principles”. Grade A: Strong Recommendation, Grade B: Recommendation, Grade C and D: option.
